# Investigating the Mechanism of Action of Anti-Dengue Compounds as Potential Binders of Zika Virus RNA-Dependent RNA Polymerase

**DOI:** 10.3390/v15071501

**Published:** 2023-07-04

**Authors:** Thamir A. Alandijany, Mai M. El-Daly, Ahmed M. Tolah, Leena H. Bajrai, Aiah M. Khateb, Isra M. Alsaady, Sarah A. Altwaim, Amit Dubey, Vivek Dhar Dwivedi, Esam I. Azhar

**Affiliations:** 1 Special Infectious Agents Unit-BSL3, King Fahd Medical Research Center, King Abdulaziz University, Jeddah 21362, Saudi Arabia; 2 Department of Medical Laboratory Sciences, Faculty of Applied Medical Sciences, King Abdulaziz University, Jeddah 21362, Saudi Arabia; 3 Department of Medical Laboratory Technology, Faculty of Applied Medical Sciences, King Abdulaziz University, Rabig 25732, Saudi Arabia; 4 Biochemistry Department, Faculty of Sciences, King Abdulaziz University, Jeddah 21362, Saudi Arabia; 5 Department of Medical Laboratory Technology, College of Applied Medical Sciences, Taibah University, Madinah 42353, Saudi Arabia; 6 Department of Medical Microbiology and Parasitology, Faculty of Medicine, King Abdulaziz University, Jeddah 20136, Saudi Arabia; 7 Computational Chemistry & Drug Discovery Division, Quanta Calculus, Greater Noida 201310, India; 8 Bioinformatics Research Division, Quanta Calculus, Greater Noida 201310, India

**Keywords:** molecular docking, RNA-dependent RNA polymerase, Zika virus, MM/GBSA, molecular dynamics simulation

## Abstract

The World Health Organization (WHO) has designated the Zika virus (ZIKV) as a significant risk to the general public’s health. Currently, there are no vaccinations or medications available to treat or prevent infection with the Zika virus. Thus, it is urgently required to develop a highly efficient therapeutic molecule. In the presented study, a computationally intensive search was carried out to identify potent compounds that have the potential to bind and block the activity of ZIKV NS5 RNA-dependent RNA polymerase (RdRp). The anti-dengue chemical library was subjected to high-throughput virtual screening and MM/GBSA analysis in order to rate the potential candidates. The top three compounds were then chosen. According to the MM/GBSA analysis, compound 127042987 from the database had the highest binding affinity to the protein with a minimum binding free energy of −77.16 kcal/mole. Compound 127042987 had the most stable RMSD trend and the greatest number of hydrogen bond interactions when these chemical complexes were evaluated further under a 100 ns molecular dynamics simulation. Compound 127042987 displayed the best binding free energy (GBind) of −96.50 kcal/mol, surpassing the native ligand binding energy (−66.17 kcal/mole). Thereafter, an MM/GBSA binding free energy study was conducted to validate the stability of selected chemical complexes. Overall, this study illustrated that compound 127042987 showed preferred binding free energies, suggesting a possible inhibitory mechanism against ZIKV-RdRp. As per this study, it was proposed that compound 127042987 could be used as a therapeutic option to prevent Zika virus infection. These compounds need to be tested in experiments for further validation.

## 1. Introduction

The Zika virus, often known as ZIKV, was first isolated from a monkey in Uganda in the year 1947. Consequently, it was investigated that the prevalence of the virus was observed in Africa and Southeast Asia. In 1954, the virus was first detected in a human in Nigeria. However, its identification was called into question and was initially assumed to be the Spondweni virus. Later, in Uganda, the first confirmed human instance of Zika was reported in 1962–1963 [1]. However, the outbreak of the Zika virus in 2007 was recorded in Africa, America, Asia, and other pacific nations [2]. After an outbreak that started in French Polynesia in 2013, its transmission was detected in Brazil for the first time in May 2015. By March 2017, 84 nations or territories throughout the world had reported autochthonous mosquito-borne Zika virus transmission, including 61 countries or territories with novel introductions of the Zika virus since the beginning of 2015 [3]. ZIKV is a mosquito-borne disease that is transmitted via the mastication of the *Aedes mosquito*, and other modes of transmission include intrauterine, sexual, perinatal, and laboratory infection and infected blood transfusion [4,5]. The infection is majorly asymptomatic, and individuals who do exhibit symptoms may experience fever, rash, muscle and joint pain, malaise, and headache, which can persist for a duration of 2–7 days. Moreover, infection with ZIKV during pregnancy was associated with congenital malformations, microcephaly, premature birth, and miscarriage. ZIKV is also linked to Guillain–Barré syndrome (GBS) and myelitis in infants and adults [6]. GBS tends to occur when the immune system erroneously assaults nerve cells in response to a viral infection. However, the pathogenesis of ZIKV infection is still under examination, although it appears to involve a combination of viral replication, immune response, genetic factors, and environmental factors [7]. Currently, ZIKV can be identified by using Zika IgM antibody capture enzyme-linked immunosorbent assays (Zika MAC-ELISA), reverse transcription quantitative real-time polymerase chain reaction (qRT-PCR), and reverse transcription loop-mediated isothermal amplification (RT-LAMP) [8].

ZIKV is a single-stranded RNA that has a total of 10,794 nucleotides and encodes a total of 3419 amino acids. It is capable of entering the host by receptor-mediated endocytosis and fusing with the endosomal cell area [9,10]. There are seven different non-structural proteins that make up the structure of ZIKV. These proteins include NS1, NS2A, NS2B, NS3, NS4A, NS4B, 2k, and NS5, the latter of which is the largest viral protein produced by ZIKV. NS5 boons as a novel antiviral target, and the protein itself is composed of three domains: A methyl transferase (MTase) domain (residues 1–262) from its N-terminal, an RNA-dependent RNA polymerase (RdRp) domain (residues 273–903) from its C-terminal, and an inter-domain region (residues 263–272) [11]. RdRp is a conserved domain that facilitates the initiation of RNA synthesis and the formation of both positive and negative RNA strands [12]. Moreover, RdRp is a versatile enzyme of RNA viruses that is essential for the replication of the genome and carries out the replication process. As stated above, the core structure of RdRp is conserved despite the divergence of its sequences. The structure of RdRp resembles a right hand that is cupped with a set of fingers, and the palm and thumbs are the sub-domain. The catalytic process of RNA-dependent RNA polymerase (RdRp) is assisted by conserved aspartates and divalent metal ions. By applying computational and experimental studies on RdRp complexes with substrates, metal ions, and inhibitors, a comprehensive understanding of their functional processes could be deciphered. Such studies provide valuable insights regarding the development of antiviral compounds [13]. Several studies showed that RdRp has the potential to be used in the development of new antiviral drugs. Similarly, Jiang et al., 2020 showed that remdesivir targets the RdRp, and due to its role as an essential enzyme for RNA replication, it can be used as potential therapeutic solution for COVID-19 [14]. Another study came to a similar conclusion, finding that the Hantaan virus (HTN)–RdRp complex should be a main focus for the development of antiviral medication. In this case, the RdRp endonuclease domain of the HTN virus possesses a catalytic activity that is dependent on a metal. The objective was to identify inhibitors capable of binding and disrupting the enzymatic activity of this metal-dependent endonuclease. For the purpose of designing inhibitors, in-computer methods such as molecular docking; molecular dynamics simulations; anticipated absorption, distribution, metabolism, excretion, and toxicity (ADMET); and drug-likeness studies were utilized [15,16].

In the current investigation, a comprehensive computational pipeline was utilized in order to search for probable hit molecules that target RdRp. To obtain the most likely conformation of the protein that could be analyzed in the MMGBSA binding free energy calculation, the protein structure was modeled, and then subsequent molecular dynamics simulations were conducted. This allowed for the generation of the most likely conformation of the protein. In this instance, the DenvInd database was compared to the three-dimensional structure of ZIKV-RdRp. The DenvInD database was developed by Dwivedi et al. and consists of compounds that have been shown to act as inhibitors against the drug targets of the dengue virus that have been validated by in vitro studies [17]. The co-crystallized ligand 4-dimethoxy-5-thiophen-2-yl-benzoic acid (G8O) and quinoline-8-sulfonamide (G8L) with ZIKV-RdRp was used as reference ligand for a comparative study. For explicit 100 ns (nanosecond) molecular dynamics simulations, the three drug-like candidates that showed the greatest promise were chosen. The active properties of the resultant protein–ligand complexes were examined using RMSD, RMSF, and MMGBSA to ascertain the hit compounds’ RdRp binding affinities.

## 2. Methodology

### 2.1. Protein Modeling

ZIKV has been recognized as a potential threat to the general population’s health. According to previous research, the RNA-dependent RNA polymerase (RdRp) found in non-structural protein 5 (NS5) of ZIKV possesses the potential to be employed as a possible target in the pursuit of drug discovery against ZIKV [18]. Consequently, the NS5 RdRp was selected as the drug target for this in silico investigation. The available crystal structure of the NS5 RdRp (PDB ID: 6LD5) was observed to comprise a solitary chain including roughly 575 residues, despite the protein sequence comprising approximately 645 residues. The protein crystal structure exhibited 70 missing residues located at various sites. In order to facilitate this structure, Swiss Model Server was employed to reconstruct the structure [19], and a complete modeled structure was used in the analysis [20]. Furthermore, the modeled structure was validated using the Ramachandran plot [21]. The stereo-chemical fidelity of the protein’s 3D model was assessed by analyzing the steric hindrance between the phi (Φ) and psi (ψ) torsion angles of amino acid residues in the Ramachandran plot [22].

### 2.2. Protein and Compound Library Preparation

The modeled 3D structure of the ZIKV-RdRp complex was used for molecular docking against the anti-dengue compound library. Here, antiviral compounds were used for virtual screening against ZIKV-RdRp and were collected from the DenvInd database [17,23]. This is an anti-dengue compound database that comprises 330 validated compounds that were used under in vitro trials as inhibitors against the respective drug target of the dengue virus (DENV). Furthermore, the database can be accessed using a web-based interface that incorporates multiple accessibility features, such as basic and advanced search options, as well as browsing functions for the data. 

The compounds were prepared using Schrodinger LigPrep [24,25]. LigPrep software facilitates the generation of 3D structures from its 2D format by including hydrogen atoms, considering bond lengths and angles, and selecting the conformer structure with the most favorable conformational energy, which is determined based on appropriate chiralities, tautomers, stereochemistry, and ring conformations. Additionally, the package employs the EPIK 2.1 ionization tool to set the ionization state within the given pH range. Moreover, the OPLS3 force field was selected for energy minimization [26].

### 2.3. Virtual Screening

In this study, structure-based virtual screening was performed using Glide extra precision (XP) of Schrodinger suite [27]. Glide XP is a computational tool used for molecular docking, which generates the docked pose and predicts the binding of small molecules to protein targets. It is a part of the Schrödinger software suite and is based on a combination of docking algorithms, including ligand conformational sampling and protein flexibility [28]. Here, the DenvInd database was used to screen anti-dengue compounds against the given protein target ZIKV-RdRp, with the aim of identifying potential binders. The process involved preparing the protein structure of ZIKV-RdRp for docking, generating conformations of the ligand database, and performing docking calculations using Glide XP. In order to determine the binding free energies of the compounds that were produced as a result of this process, molecular mechanics/generalized Born surface area (MMGBSA) was used as a method of analysis. The compounds were rated according to the free energy that they contributed to the binding process, and the top three hits were chosen for more research. Afterwards, simulations of molecular dynamics were utilized to determine the degree of flexibility and stability possessed by these molecules.

### 2.4. Molecular Dynamics Simulation

The molecular dynamics simulation will now begin with these three molecules as the starting point. In order to choose the docked pose for the top three hits, the Maestro-Desmond version 5.6 module of the Schrodinger Master version 11.8 suite was used to run a molecular dynamics (MD) simulation for a duration of 100 nanoseconds [29]. The protein was prepared using the Schrodinger suite’s protein preparation wizard module using evasion parameters. The system building tool was used to produce an orthorhombic box to utilize as the simulation box. Within a 20-angstrom radius of the ligand binding sites, both salt and ion placement were avoided as much as possible. The complete apparatus was submerged in a water bath containing sodium counterions and a model of water based on the TIP4P system. A 20 psi NPT reassembly was performed after the MD simulation was completed with a constant pressure of 1 atm utilizing an anisotropic diagonal position scaling of 0.002 ps time steps. The compactness of the system was kept at 1 gram per cubic centimeter throughout. For the molecular dynamics (MD) calculations, the programs Desmond version 5.6 and Maestro Suite version 11.8 with the OPLS-2005 force field were utilized. In order to achieve representative conformations, the obtained trajectories were clustered using the Desmond Trajectory Clustering tool, which was developed in Maestro Suite. The clustering was performed based on RMSD.

### 2.5. MM/GBSA Calculation

For the purpose of determining the total binding free energies (Δ*G_Bind_*) of ligands, the Schrodinger Maestro Prime module, along with the OPLS-2005 force field force that was utilized for in silico docking, and the Prime MM-GBSA module were utilized [30,31]. The energy of optimized free receptors, a free ligand, and a complex of the ligand with a receptor was calculated by Prime MM-GBSA. In addition to this, it computed the ligand strain energy by dispersing the ligand throughout a solution that was automatically produced by the VSGB 2.0 suit [32]. In this case, the input structures that were used in the calculations were acquired from the 100 ns MD simulation trajectory of each protein–ligand system. These structures were then employed in the computations. In this case, the evaluation of the total binding free energies of the top three hits was carried out using only the final 10 ns of the trajectory. In addition to this, the trajectories of the native complex over the previous ten nanoseconds were utilized in the computation of the total binding free energy. Using Equation (1), we were able to compute the net free binding energy, also known as *G*. This allowed us to estimate the individual energy components of the protein (receptor), the ligand, and the protein–ligand complex.

(1) $$\Delta G_{Bind}=\Delta G_{complex\;\left(minimized\right)}-\left(\Delta G_{protein}+\Delta G_{ligand}\right)$$

In Equation (1), $\Delta G_{Bind}$ represents the change in total binding free energy, $\Delta G_{complex\;\left(minimized\right)}$ represents the change in binding free energy of the complex, $\Delta G_{protein}$ shows the change in binding free energy of the receptor, and $\Delta G_{ligand}$ indicates the change in binding free energy of the ligand. Eventually, the simulation trajectory was converted into a Bio3D-compatible format [33,34] to perform the principal component analysis (PCA) [35]. R was employed in this application. Here, the molecule’s initial coordinate was applied as a point of reference, and subsequent conformations generated using the simulation were superimposed onto it to determine the eigen vector. This vector is indicative of the molecule’s orthogonal principal components of motion.

## 3. Results and Discussions

### 3.1. Protein Model Validation

**Figure 1:**
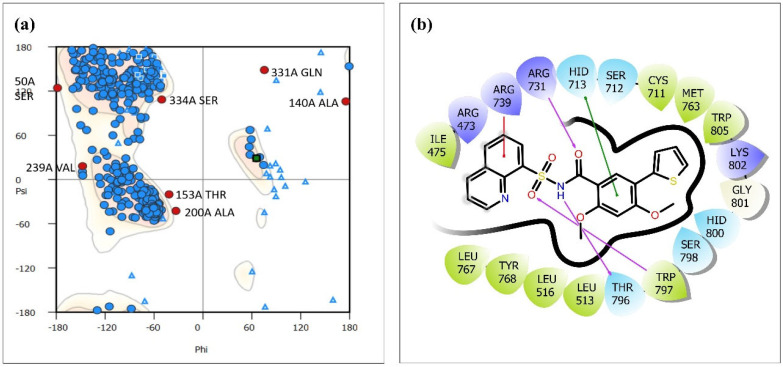
(**a**) Ramachandran plot of modeled RdRp protein of ZIKV (6LD5) and (**b**) interaction plot of native ligands 2,4-dimethoxy-5-thiophen-2-yl-benzoic acid (G8O) and quinoline-8-sulfonamide (G8L) with protein RdRp.

The protein RdRp of ZIKV was retrieved from the PDB database with PDB ID 6LD5. There were 70 missing residues at different sites within the protein crystal structure, and the SWISS-MODEL server was used to remodel the structure for further experiments. Residues 248 to 269, 314 to 320, 344 to 348, 407 to 426, 461 to 471, and 888 to 891 were found to be missing in the crystal structure and were added during the remodeling of the protein structure. The remodeled structure was validated using the Ramachandran plot, as shown in Figure 1a, in which ~92% of the residues were found to be within the allowed region. Seven residues turned out to be outliers; however, those were originally a part of the experimentally obtained crystal structure and were therefore left undisturbed. The crystal structure obtained from PDB had binding sites for zinc ions and two native ligands, namely, 2, 4-dimethoxy-5-thiophen-2-yl-benzoic acid (G8O) and quinoline-8-sulfonamide (G8L). The two ligands were bound to the crystal structure by four hydrogen bonds and 12 non-bonded contacts, as shown in Figure 1b. Moreover, the two native ligands were also found to be covalently bound to each other while occupying a region around the allosteric N-pocket of the protein. This validated, modeled protein was used further as a target protein structure for molecular docking.

### 3.2. Virtual Screening and MM/GBSA (Δ*G* Binding Free Energy)

Virtual screening is a computational approach that employs an extensive and heterogeneous collection of chemical compounds to identify potential drug-like molecules [36]. In this study, 330 molecular structures were used in the screening. Schrodinger Glide XP (Schrödinger Release 2023-1: Glide, Schrödinger, LLC, New York, NY, USA, 2021) was used for the virtual screening of these compounds [27]. The twelve compounds with the most superior docking scores, which exhibited the most optimal interactions within the protein’s native ligand binding pocket, were selected for further examination. The docking scores for the twelve selected compounds, which ranged between −10.23 kcal/mol and −12.95 kcal/mol, were significantly higher than that of the native compound complex, which scored −4 kcal/mol. These scores were negatively correlated with the reference native ligand. Previous studies with similar in silico approaches on ZIKV-RdRp showed inhibitor compounds with docking scores ranging from −6.13 kcal/mol to −9.20 kcal/mol, which was higher than the top twelve selected compounds in this study [37,38,39]. The docking score of the native ligand was lower than the selected ligand, which suggested a higher strength of binding, but the docking program is a rigid protocol, and, thus, it has limitations in finding the optimum minimum energy state. This was later addressed by running the molecular dynamics simulation. Moreover, MMGBSA is a stronger categorization system; thus, it can be used to assess a protein–ligand binding affinity for docked complexes.

**Figure 2:**
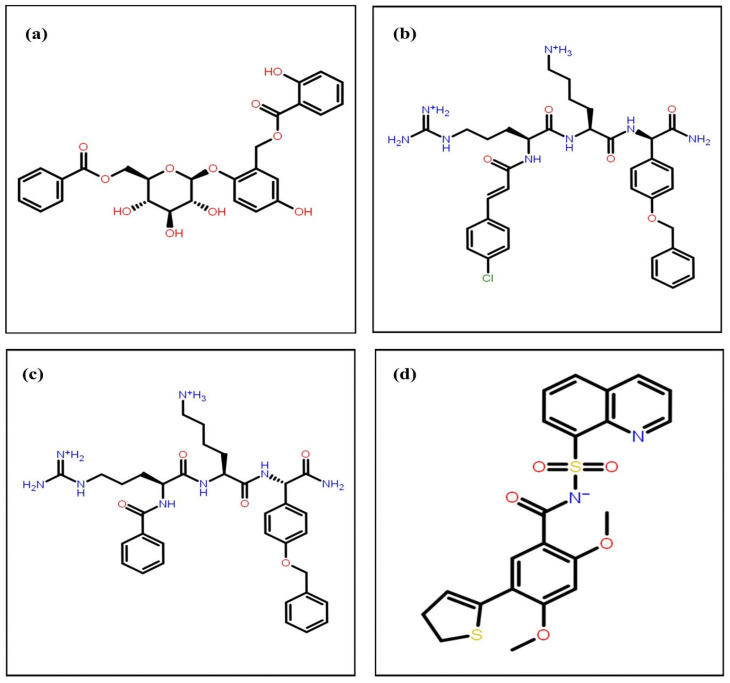
2D structure representation of the selected top hit compounds based on the MMGBSA binding energy with DenvInd database IDs (**a**) 44577154, (**b**) 127040817, (**c**) and 127042987 and the co-crystallized native ligand (**d**) (G8O-G8L).

Moreover, an MM/GBSA (molecular mechanics/generalized Born surface area solvation) study was conducted for calculating the Δ*G* binding energy for the selected compounds using the Prime tool to validate the top three hit compounds to be considered for further analysis. Δ*G* binding energy (Δ*G*) is based on the binding free energy between ligands and proteins and is considered to be more accurate in predicting the strength of protein–ligand interactions compared to docking scores [40]. In addition to the docking scores, Table 1 showed a comparative analysis of the binding free energy (Δ*G*) for the twelve selected compounds that resulted from the virtual screening, as well as the native ligand. The Δ*G* binding energy of the native ligand was −45.85 kcal/mol, and all the ligands, except for ligand 127038864 (Δ*G* = −42.46 kcal/mol) and ligand 71455121 (Δ*G* = −41.09 kcal/mol), had a negatively higher Δ*G* binding energy than the native ligand. Ligand 127042987 had the most negative Δ*G* binding energy score of −77.16 kcal/mol, while ligands 127040817 and 44577154 had Δ*G* binding energy scores of −71.88 kcal/mol and −68.22 kcal/mol, respectively. These three ligands, which had the most negative Δ*G* binding energy scores, exhibited the highest binding affinity with the protein and were preferred for further analysis during the study. Figure 2 shows the two-dimensional structures of the best three compounds with the native compound. Even in the MMGBSA binding energy calculation, the ligands selected in the screening showed a greater binding strength with the protein molecule in comparison to the native ligand. However, in addition to the binding score, interactive residues that participate in the protein–ligand interaction also determine the inhibitory mechanism of the proposed compounds. The total number of interactions with their type indicate the strength of binding. Moreover, direct interaction with the catalytic residues could suggest their inhibitory action. In the next phase of the study, the interactions were studied between compounds (screened ligands and native) and proteins.

### 3.3. Molecular Dynamics (MD) Simulation

**Figure 3:**
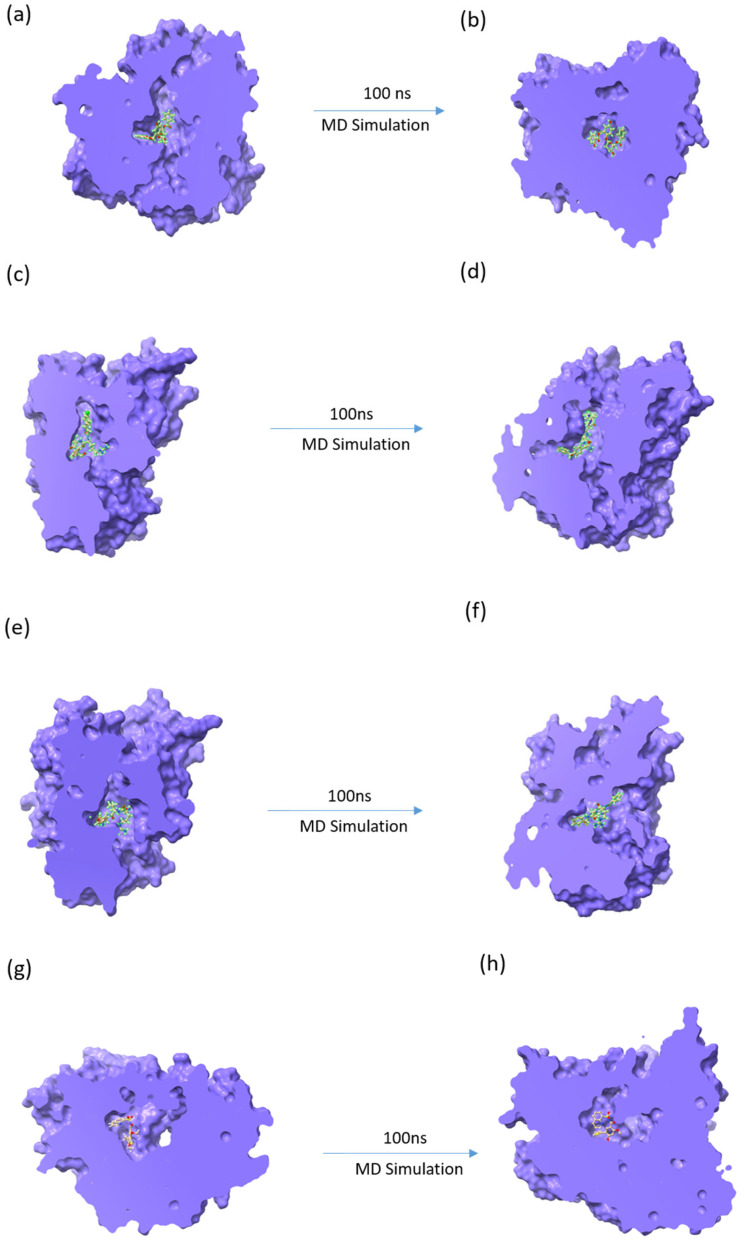
First and last pose of the protein–ligand complexes after the 100 ns MD simulation for (**a**,**b**) 44577154, (**c**,**d**) 127040817, (**e**,**f**) 127042987, and (**g**,**h**) the native ligand (G8O-G8L).

In recent years, molecular dynamics simulations have developed into more advanced methods that can be successfully applied to the process of comprehending the structure of biological macromolecules [41]. Here, in addition to the 100 ns simulation, two more replicas were performed to ensure the accuracy and robustness of the outcomes. Here, Figure 3 shows the first and last poses during a 100 ns protein–ligand MD simulation study. It was observed that during the entire MD simulation run, the ligands were bound to the protein within the binding pocket. Although fluctuations and conformational changes were observed in the ligands when first and last poses were compared, as observed in Figure 3, all three ligands remained in the bound state throughout the simulation. Similar observations were made for the protein–native ligand MD simulation. This indicates that the ligand forms stable contact with the protein even in its native environment. However, both rotational and translational motion was seen in all the ligands in their last pose of the MD simulation, which was also observed in the protein–native ligand complex. Furthermore, various MD analyses were performed for a better understanding of the protein–ligand complex stability and interactions.

#### 3.3.1. RMSD (Root-Mean-Square Deviation)

**Figure 4:**
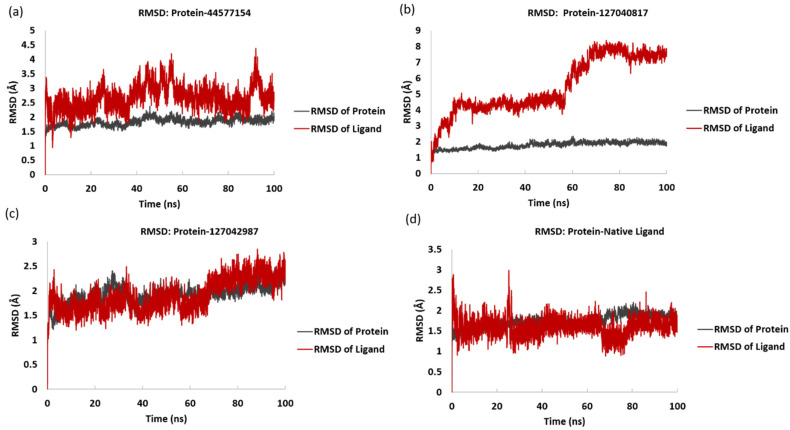
Root mean square deviation (RMSD) of the protein and ligand for the selected protein–ligand complexes after the 100 ns MD simulation for (**a**) 44577154, (**b**) 127040817, (**c**) 127042987, and (**d**) the native ligand (G8O-G8L).

RMSD, or root-mean-square deviation, indicates the average deviation of a structure from its reference frame (equilibrated) during the simulation. During RMSD calculations, the protein backbone was taken as a reference frame, and changes within the protein backbone and ligand bound to the protein were plotted separately. The RMSD helps with insights into the structural or conformational changes within the protein and the bound ligand throughout the duration of the simulation. Here, if the RMSD values stabilize over a certain value during the simulation, it indicates that the protein–ligand complex has reached a state of either local or global minimum energy, and a stable equilibrium is achieved. However, if the RMSD values keep increasing, this indicates that the complex has not yet stabilized, and the local or global minimum energy values for the protein–ligand complex have not been achieved. RMSD values in the range of 1–3 Å are considered acceptable, especially for small and globular proteins [42]. If the RMSD values are found to fall outside this range, it is indicative of a large conformational change within the structure. The lower RMSD value throughout the MD simulation suggests that the protein–ligand complex is more stable, whereas a higher RMSD value indicates that the protein–ligand complex is somewhat less stable [43,44]. Figure 4 shows the RMSD of the protein and ligand for all four systems. In the protein–native ligand system, it was observed that the mean RMSD for the protein backbone when bound to the ligand was found to be 1.76 Å, and the mean RMSD for the ligand bound to the protein was found to be 1.59 Å, which is well within the range of 1–3 Å. Although a few sharp peaks were observed in the RMSD values of the ligand during the 20–30 ns timeframe, the ligand RMSD stabilized after 30 ns and sustained until 65 ns in the MD simulation. A decrease in the ligand RMSD values below the mean RMSD of the ligand was observed between 60 ns and 80 ns. In the last 20 ns, the RMSD values remained stable and consistent with the mean RMSD value of the ligand. On the other hand, the protein backbone was observed to be more stable, and a stable equilibrium was achieved at 11 ns and was sustained until 50 ns. Further, the RMSD values for the protein backbone decreased below the mean RMSD of the protein, and after 69 ns, they increased slightly above the mean RMSD value and sustained until 100 ns in the MD simulation. This indicates that the protein backbone was found to be comparatively more stable and that the RMSD values were well within the acceptable range of 1–3 Å. Based on the RMSD values of the protein–native ligand complex, it can be concluded that the native ligand binds strongly to the protein even in the native environment of the protein. It can also be concluded that the ligand binding does not bring any significant change in the protein conformation. The MD simulation analysis results for the protein–native ligand complex were compared with the results for the protein and the top three ligands (PubChem Id: 127042987, 127040817, and 44577154). The mean RMSD values for the protein were found to be 1.82 Å, 1.78 Å, and 1.91 Å when bound to ligands 44577154, 127040817, and 127042987, respectively, which is comfortably within the acceptable range of 1–3 Å. On the contrary, the mean RMSD values for the ligands were found to be 2.66 Å, 5.47 Å, and 1.90 Å for ligands 44577154, 127040817, and 127042987, respectively, when bound to the protein. It was observed that the mean RMSD for ligand 127040817 was significantly larger than the protein's RMSD, which indicates that the ligand shifted from its initial binding position to occupy a different binding position. This result is in accordance with the first and last pose data for ligand 127040817, which shows a change in conformation, and there are possibilities that the ligand shifted to a new binding location that was different from its initial binding location. The mean RMSD for ligand 127042987 was found to be very close to the mean RMSD of the protein during the simulation. This indicates that the ligand consistently occupied the initial binding site till the end of the simulation, which is at 100 ns. The protein’s RMSD values when bound to all three ligands revealed consistent stability throughout the simulation. This indicates that the protein was stable during simulation when bound to all of the three ligands. The protein RMSD was observed to be in accordance with the RMSF data for the protein.

The other two replicas of the 100 ns simulation were also used for the calculation of RMSD to remove the bias caused by the initial velocity imparted during the simulation. Supplementary Figure S1 shows the RMSD of the protein and ligand for the three complexes. Here, it was observed that the RMSD was the same as the first 100 ns simulation trajectory, ensuring that the outcomes were reproduced in all three trajectories. This provided additional confirmation of the robustness of the simulation results.

#### 3.3.2. RMSF (Root-Mean-Square Fluctuation)

**Figure 5:**
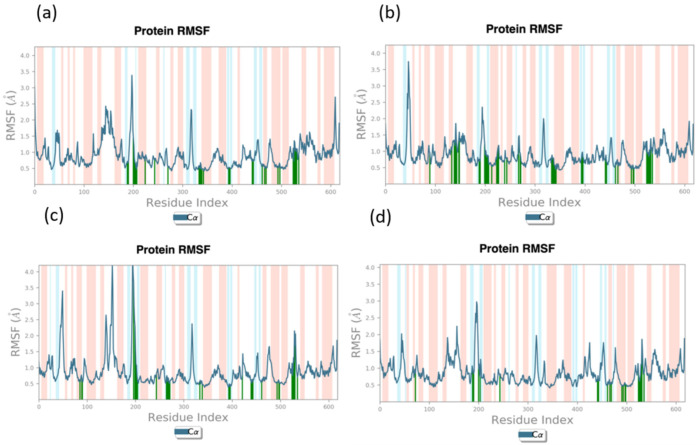
Root-mean-square fluctuation (RMSF) of the protein in the protein–ligand complexes for each atom averaged over the 100 ns MD simulation for (**a**) 44577154, (**b**) 127040817, (**c**) 127042987, and (**d**) the native ligand (G8O-G8L).

RMSF, or root-mean-square fluctuation, exhibits the average fluctuations within each residue of the protein over the course of the simulation. It was observed that, except for a few residues, the residues of the protein exhibited RMSF values less than 2.5 Å, as shown in Figure 5, and the RMSF values for the three hit ligand systems were comparable with that of the native ligand system. This indicates that fluctuations exhibited by the residues at their loci were not very significant, and the overall protein was found to be quite stable, as also indicated by the RMSD values. Here, the protein for the native ligand complex showed all residues with RMSFs < 3 Å, while there were two residues with RMSFs > 3 Å for compounds 44577154 and 127040817, although these residues were not in contact with ligand atoms. However, for ligand 127042987, it was found that six residues had RMSFs > 3 Å, and these residues, except for one (Phe^466^), had no contact with the compound atoms.

The RMSFs of the other two replicas were also observed, as shown in Supplementary Figure S2. It was found that the RMSF was the same for the replicas, as observed in the first 100 ns simulation, showing that the results were reproduced accurately.

#### 3.3.3. Protein–Ligand Interactions (MD Simulation)

**Figure 6:**
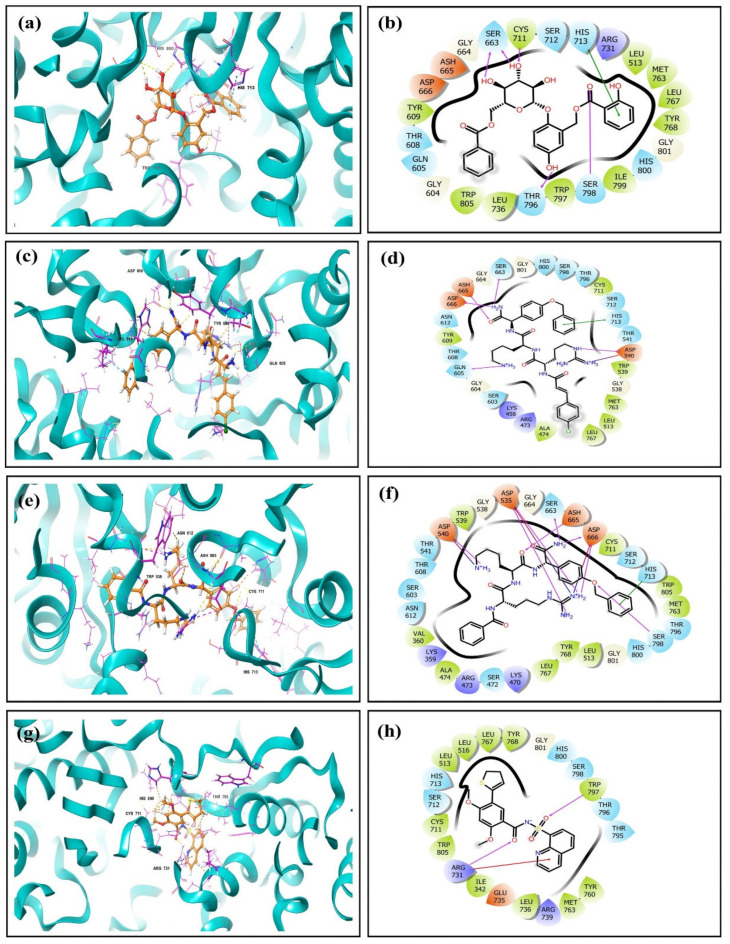
3D conformation of ligands and molecular interaction in a 2D representation for (**a**,**b**) 44577154, (**c**,**d**) 127040817, (**e**,**f**) 127042987, and (**g**,**h**) the native ligand (G8O-G8L).

Here, Figure 6 showed the three top-ranked compounds that resulted from the docking study, along with their corresponding molecular interactions with the protein molecule at the binding site. In this case, various molecular interactions were observed that include hydrogen bonds (H-bonds) to hydrophobic contacts, π-cation interactions, π-π stacking, and salt bridges. In this study, ligand 44577154, shown in Figure 6a,b, was found to form five hydrogen bond interactions and single π-π stacking with surrounding residues. The distance between the hydrogen bond donor and acceptor is shown with the name of the H-bond-forming residues to indicate the strength of the H-bond. Specifically, the Cys^711^ (3.52 Å), Ser^798^ (1.9 Å), and Thr^796^ (1.9 Å) residues were involved in the formation of single hydrogen bonds, while Ser^663^ (2.8 Å and 2.1 Å) formed a double hydrogen bond with the ligand. Moreover, the His^713^ residue displayed a stacking interaction with the ligand. Ligand 127040817, represented in Figure 6c,d, was found to be walled by various residues. Specifically, Asp^540^ (1.9 Å) formed a single hydrogen bond and salt bridge; Ser^663^ (2.1Å), Asp^666^ (2.99 Å), Ash^665^ (2.0 Å), and Gln^605^ (2.5 Å) each formed a single hydrogen bond; and, lastly, His^713^ showed stacking. These are categorized as weak hydrogen bonds. Ligand 127042987 formed individual hydrogen bonds with the Ser^663^ (1.7 Å), Ash^665^ (1.7Å), Asp^666^ (2.1 Å), Asp^540^ (2.4 Å), and Ser^798^ (2.1 Å) residues, as well as salt bridges with the Asp^540^ and Asp^666^ residues. In addition, stacking was observed between the ligand and the His^713^ residue, as shown in Figure 6e,f. In contrast, the native ligand used as a control for the experiment exhibited only two hydrogen bonds that include residue Arg^731^ (3.2 Å) and Trp^797^ (2.1 Å), while Arg^731^ also showed a π-cation interaction with the ligand, as shown in Figure 6g,h. The comparative study showed that the residue binding interactions in the native ligand were not observed in the top three selected ligand complexes. This indicates the binding of the ligand at a different binding cavity compared to the native ligand. In the co-crystallized structure (PDB ID:6LD5), the ligand showed H-bonds with Thr^796^, Arg^731^, and Trp^791^. These interactions matched the docked complex of the native ligand and protein, missing the Thr^796^ H-bonds. This specific interaction was also observed in ligand 44577154, which indicates interactions similar to those of the native ligand. Similar interactions were detected for potential inhibitors in previous studies in which ZIKV-RdRp was targeted and residues Cys^711^, Arg^731^ and Asp^666^ showed interactions after molecular docking [37,45]. Figure 3 shows that native interacting residues were also detected in the hit compound complexes, but their distances were not optimum for forming the hydrogen bonds. As discussed above, these complexes were generated in the rigid docking, so there is a high possibility that the initial pose could change, and ligands can transition to the minimum energy and maximum interaction state. In this regard, the best docked pose complexes of the top three hits were used for studying their stability with the molecular dynamics simulation. Here, the ligand could move in the binding site to achieve the optimum interaction.

Later, the simulation trajectory was used to map the interactions and their corresponding changes to understand the stability of the protein–ligand complex. In the course of studying the protein–ligand interaction, ionic interactions were observed to have the highest bond strength; however, weaker interactions also play a crucial role in a physiological system due to their tunability and their mediation by affinity and concentration. Therefore, while considering the protein–ligand interaction, apart from just the strength of the interactions, the type of interaction is also critical to understand the interplay between the ligand and the protein under a physiological condition.

**Figure 7:**
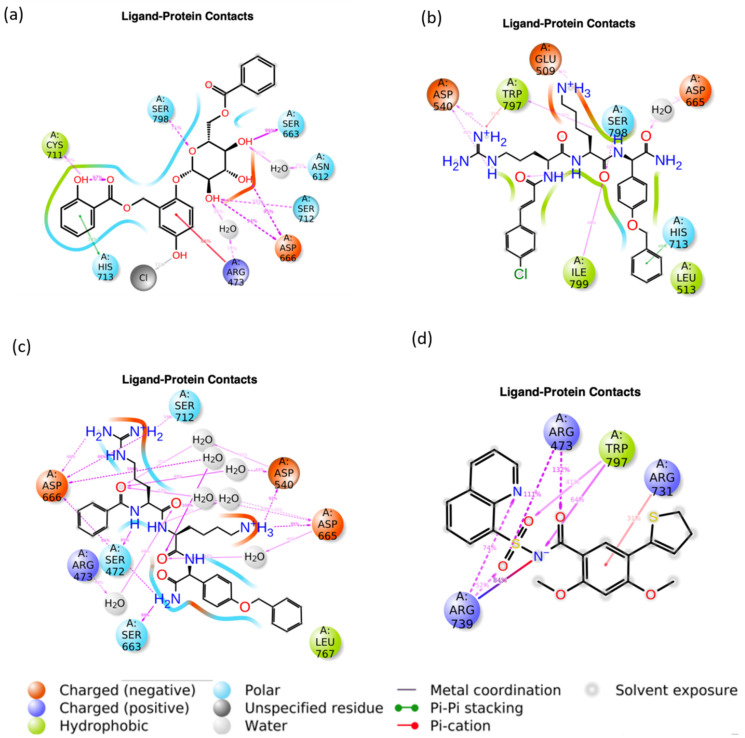
Intermolecular interaction mapping during the 100 ns MD simulation for (**a**) 44577154, (**b**) 127040817, (**c**) 127042987, and (**d**) the native ligand (G8O-G8L).

Figure 7 displays the 2D representation that was taken from the frames created during the 100 ns MD simulation of the interaction between the ligand and the protein. This chart displayed the percentage of frames that were associated with each form of interaction. In this investigation, the cut-off was determined to be 50%, which indicates that the specified bonds must be seen 50% of the time before the interaction can be regarded as a stable type. During the MD simulation analyses, as depicted in Figure 7d, it was observed that the native ligand formed hydrogen bonds with four residues, namely, Arg^473^, Arg^731^, Arg^739^, and Trp^797^. Here, Arg^473^ and Arg^739^ had two hydrogen bonds with the native ligand, with more than 50 % of the timeframe of the simulation. Here, it is notable that Arg^731^ and Trp^797^ are the native interaction detected in the crystallized structure and retained during the MD simulation. However, Trp^797^ had only one hydrogen bond with >50% occupancy, while Arg^731^ had π-cation interaction with 31% of occupancy. A salt bridge was also observed between the protein–native ligand complexes with 84% of the timeframe of simulation. Comparatively, the binding interaction of the docked complex showed that only Trp^797^ was retained during the MD simulation, while Arg^731^, which formed a hydrogen bond, converted to a π-cation interaction with less than 50% occupancy. In contrast, ligand 44577154 formed hydrogen bonds with seven residues of the protein, namely, Asn^612^, Ser^663^, Asp^666^, Cys^711^, Ser^712^, Arg^473^, and Ser^798^, while His^713^ formed a π-π stacking interaction, and Arg^473^ formed a π-cation interaction with 39% and 66% of the total time frame, respectively. Here, Ser^663^, Ser^712^, and Ser^798^ had one hydrogen bond each, whereas Arg^666^ had two hydrogen bonds with > 50% occupancy, as shown in Figure 7a. Based on the binding interactions of the docked complex (ligand 44577154), it was found that Ser^663^ and Ser^798^ retained the hydrogen bond interactions with more than 50% occupancy during the MD simulation. Figure 7b showed that ligand 127040817 had hydrogen bond interactions with six different residues, Glu^509^, Asp^540^, Asp^665^, Ser^798^, Trp^797^, and Ile^799^, while Leu^513^ showed hydrophobic contact, and Trp^797^ and His^713^ showed π-cation and π-π stacking, respectively. However, there were no residues with more than 50% occupancy, while Asp^540^, Ser^798^, and Ile^799^ had occupancies of 49%, 50% and 48%. In comparison with the interactions of the docked protein–ligand complex, only Asp^665^ retained its hydrogen bond with the ligand during simulation but with 30% occupancy. Ligand 127040817 had the highest number of hydrogen bonds compared to the other compounds. As shown in Figure 7c, the ligand had multiple hydrogen bond interactions with Ser^472^, Asp^540^, Arg^473^, Ser^663^, Asp^665^, Asp^666^, and Ser^712^, all of which except Arg^473^ had occupancies > 50%, while Arg^473^ had an occupancy of 49% for the total timeframe. Compared with the docked complex of the protein–ligand (127040817), the hydrogen-bond-forming residues Asp^665^, Asp^666^, Asp^540^, and Ser^663^ were retained during the MD simulation of the complex with more than 50% occupancy. It was interesting to note that, for some of the residues, the changes from the docked pose in the protein–residue interaction with ligand 127042987 was lesser compared to the other compounds, including the native ligand. Here, ligand 127042987 retained most of the hydrogen-bonding residues during the MD simulation, which suggests that the bonds formed by ligand 127042987 are more stable than the bonds formed by ligand 44577154 and ligand 127040817.

Intermolecular interaction mapping was also performed for the other two replicas, as shown in the Supplementary Figure S3. It was observed that the interactions in the two replicas were up to the 100 ns simulation trajectory discussed here. This ensured the reproducibility of the results during the simulation.

From the interaction analysis data, it can be inferred that the atoms of ligand 127042987 interacted more strongly with the protein residues when compared to the other two test ligands, 44577154 and 127040817. Based on the MD simulation results, ligand 127042987 can be concluded to act as a promising potential drug molecule when compared to the ligands 44577154 and 127040817. In order to confirm the results obtained from the MD simulation, an MM/GBSA study was performed to evaluate the Δ*G* binding free energy.

#### 3.3.4. SASA (Solvent Accessible Surface Area)

**Figure 8:**
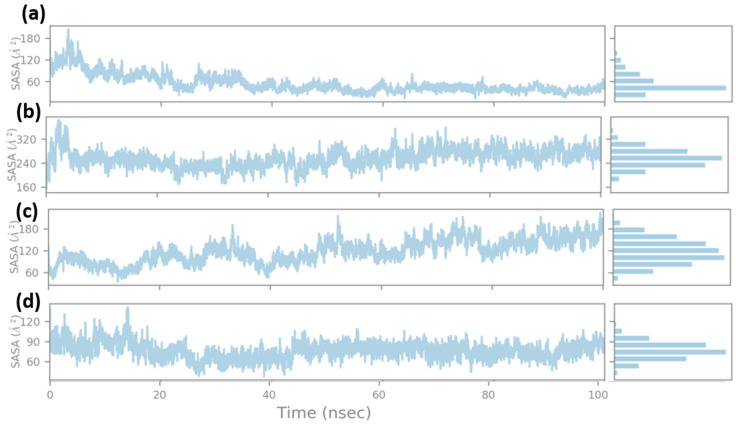
SASA (solvent accessible surface area) during the 100 ns MD simulation for (**a**) 44577154, (**b**) 127040817, (**c**) 127042987, and (**d**) the native ligand (G8O-G8L).

The SASA of the ligand molecule was calculated for the 100 ns simulation. As shown in Figure 8, the solvent accessible surface area (SASA) of the ligand indicated the surface area of the ligand that was exposed to the solvent. Here, the native ligand showed a SASA of 90 Å^2^ for most of the simulation time frame, while it had few fluctuations when SASA reached 120 Å^2^. Compound 44577154 showed comparatively higher SASA with a magnitude from 120 Å^2^ to 180 Å^2^ at the initial phase during the MD simulation, which gradually decreased to 60 Å^2^ for most of the simulation frames. Compound 127042987 had the lowest SASA, with 60 Å^2^ at the beginning of the 100 ns simulation, which indicated that less of the ligand’s surface area was exposed to the solvent, similarly to the binding surface of the protein. However, the SASA increased to the range of 120 Å^2^ to 180 Å^2^ for the later state of the simulation. In contrast, compound 127040817 had a relatively higher SASA of 320 Å^2^ with a higher fluctuation, while most of the frames had a SASA of 240 Å^2^. However, the solvation of the compounds was higher compared to the native ligand.

The SASA from the other two replicas of the 100 ns simulation was also calculated as shown in the Supplementary Figure S4. It was observed that the ligands had a similar SASA trend to the first 100 ns simulation. Here, 127040817 and 127042987 showed the same behavior in SASA, while 44577154 showed a marginal change. Ligand 44577154 had a SASA of 120 Å^2^ to 160 Å^2^ at the initial phase during the MD simulation; however, it gradually decreased to 80 Å^2^ for most of the simulation. Running the simulation in triplicate showed similar results in SASA, which confirmed the accuracy of the simulation outcome.

## 4. MM/GBSA Analysis (Δ*G* Binding Free Energy)

The binding free energy (Δ*G*) using the MM/GBSA approach has been found to be more reliable and replicable as it takes into consideration various physical and chemical parameters for determining the most stable conformation with the highest negative energy. Here, the calculation of Δ*G* binding free energy was performed on the final 10 ns of each trajectory, resulting in 500 frames, which means the energy was calculated after every 20 ps. Similarly, the standard deviation of the binding free energy was calculated for the last 10 ns of simulation trajectory. The results of the MM/GBSA are shown in Table 2. The protein–ligand complex obtained from the MD simulation study was subjected to the Δ*G* binding free energy study, and it was observed that the Δ*G* binding free energy for ligand 127042987–protein complex was found to possess the highest negative energy, which was −96.50 kcal/mol. It was also significantly higher than the native protein–ligand complex, which was found to be −66.17 kcal/mol. The other two ligand (ligands 44577154 and 127040817) complexes were found to possess a Δ*G* binding free energy of −68.77 kcal/mol and −89.46 kcal/mol, respectively. Earlier studies using MM/GBSA to evaluate potential inhibitors of ZIKV-RdRp showed higher binding free energy (−25.04 kcal/mol) results compared to this study, indicating that the selected top three hits had strong binding [46]. The results suggest that ligand 127042987 can act as the most potential molecule for acting as a drug against Zika virus. The data in Table 2 also suggest that the 127042987–protein complex had minimum fluctuation in the MMGBSA score, which additionally confirms the stable binding of the ligand with the protein.

## 5. Principal Component Analysis

**Figure 9:**
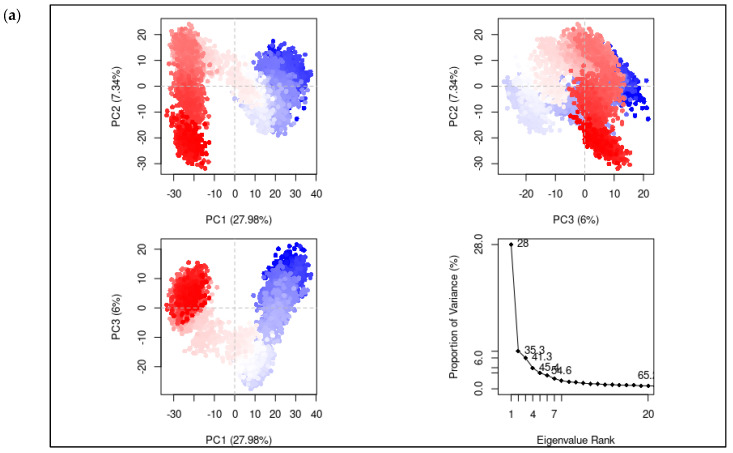

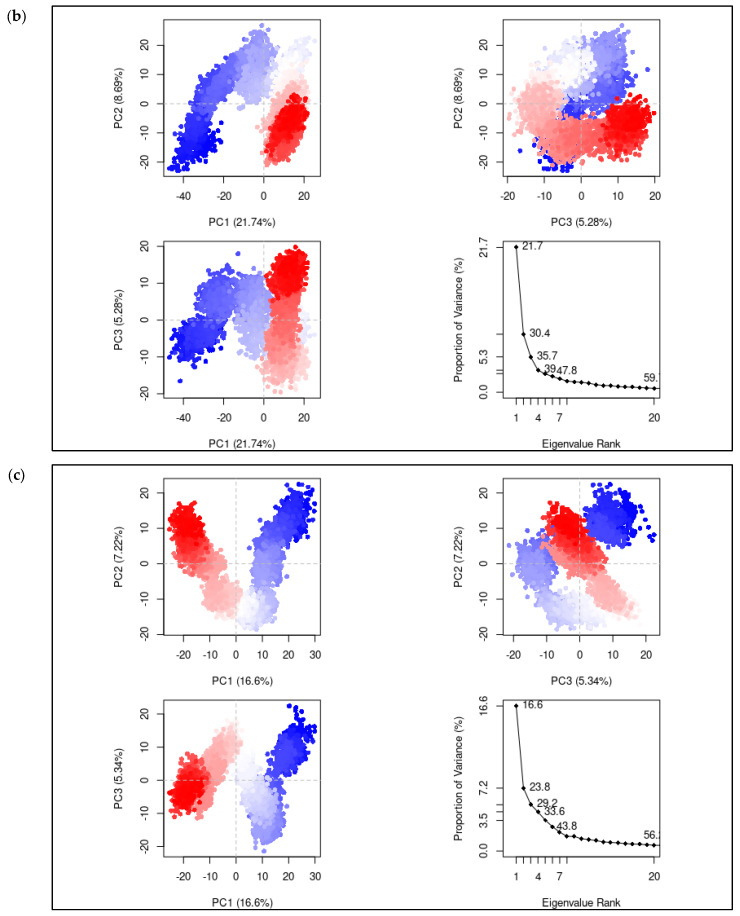

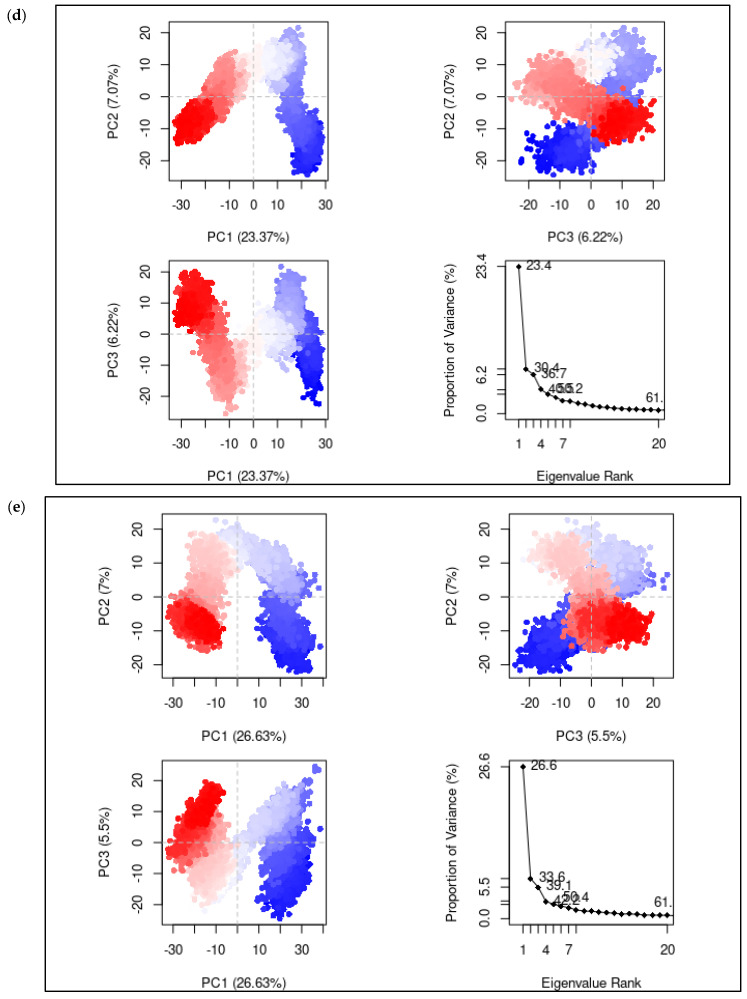
PCA analysis of (**a**) the apo form of the protein, (**b**) the bound control ligand, (**c**) 44577154, (**d**) 127040817, and (**e**) 127042987.

Principal component analysis (PCA) was employed to assess the impact of ligand binding on the conformational dynamics of the protein in its unbound (apo) and bound forms. The PCA encompassed the entire 100 ns trajectory, focusing exclusively on the Cα atoms of the protein. The subfigures presented in Figure 9 illustrate the conformational changes observed in the protein as a result of ligand binding. To establish a reference for acceptable motion upon ligand binding, a control ligand (Figure 9b) was included in the PCA. The covariance matrix’s Eigen vectors represented the principal components of motion. The first principal component of the unbound protein conformation accounted for 27.98% of the overall motion. Subsequently, the second (PC2) and third (PC3) components contributed 7.34% and 6%, respectively (Figure 9a). In comparison, the control ligand bound to the protein exhibited variances of 21.74%, 8.69%, and 5.28% in its top three principal components (Figure 9b). These results indicate a reduction in the degree of freedom upon ligand binding as the variance in the principal components decreased for the ligand-bound protein. The principal components of motion for the top three hit compounds (Figure 9c–e) demonstrated a similar trend, indicating the restrained motion of the Cα atoms in the protein upon binding with these compounds. The binding event restricts the movement and flexibility of the protein, leading to a decrease in the available degrees of freedom. This loss of entropy is often associated with the formation of specific interactions between the ligand and the protein, such as hydrogen bonds, electrostatic interactions, and hydrophobic interactions. These interactions stabilize the binding conformation and limit the protein’s conformational space, resulting in a decrease in entropy. Compound 44577154 brought the maximum loss of entropy (PC1 = 16.6) compared to the unbound state (PC1 = 27.98%). This indirectly also showed the stronger binding of compound 44577154 with the protein, which restricted the conformational motion of the protein. The reduction in entropy upon ligand binding is a crucial factor in determining the thermodynamics and energetics of the binding process. It contributes to the overall affinity and specificity of the protein–ligand interaction. By constraining the protein’s motion and promoting a more organized state, ligand binding facilitates the formation of a stable complex and promotes specific biological functions or signaling pathways.

## 6. Conclusions

This study targets the RNA-dependent RNA polymerase (RdRp) of Zika virus to inhibit its growth by deactivating its enzymatic function. The known ligand, G8O-G8L, which is co-crystallized with the protein RdRp was used as a reference in this investigation to compare the outcome. The target ZIKV NS5 RdRp was subjected to computational screening with anti-dengue compounds acquired from the DenvInd database. The docked scores of compounds were compared with the reference ligand. Virtual screening resulted in the selection of 12 compounds followed by Δ*G* binding free energy calculation to select the top three hits for further investigation. In the MD simulation analysis of the top three complexes, the RMSD and RMSF established that ligand 127042987 had higher stability comparatively. The MM/GBSA analysis on the trajectory reconfirmed the results using Δ*G* binding free energy that showed that ligand 127042987 had the most stable complex; thus, this compound should be taken further for in vitro studies to validate its inhibitory action. Overall, this study proposed a possible inhibitor against Zika NS5 RdRp based on computational simulations.

## Figures and Tables

**Table 1:** Top 12 hit compounds from the DenvInd database screened against the RNA-dependent polymerase of Zika virus.

Compounds	Docking Score (kcal/mol)	MM/GBSA Δ*G* Binding Free Energy (kcal/mol)
127042987	−10.52	−77.16
127040817	−10.46	−71.88
44577154	−10.60	−68.22
127040514	−10.96	−64.52
57409245	−12.95	−63.77
127038506	−10.46	−63.56
57409246	−11.52	−60.01
127038036	−10.23	−58.23
127040814	−10.97	−50.03
118717692	−12.18	−47.56
127038864	−10.58	−42.46
71455121	−10.90	−41.09
Native (G8O-G8L)	−4.00	−45.85

**Table 2:** Average total binding free energy (kcal/mol) values computed by molecular mechanics/generalized Born surface area (MM/GBSA) for ZIKV-RdRp, docked with the screened top three compound complexes and the native ligand G8O-G8L complex.

MM/GBSA Components(kcal/mol)	44577154	127040817	127042987	Native Ligand
Δ*G_Bind_*	−68.77 ± 6.399	−75.49 ± 7.97	−96.50 ± 3.29	−66.17 ± 3.39
Δ*G_Bind_* Coulomb	−34.41 ± 3.91	−21.41 ± 24.27	−56.98 ± 35.78	−32.82 ± 17.65
Δ*G_Bind_* Covalent	1.94 ± 2.20	4.72 ± 2.13	6.68 ± 1.92	1.78 ± 2.55
Δ*G_Bind_* Hbond	−4.22 ± 0.34	−2.58 ± 0.73	−7.16 ± 0.40	−3.89 ± 0.22
Δ*G_Bind_* Lipo	−17.56 ± 2.14	−26.48 ± 2.81	−26.58 ± 0.63	−20.08 ± 0.60
Δ*G_Bind_* Solv GB	49.09 ± 3.55	46.13 ± 25.21	60.67 ± 35.14	46.29 ± 17.33
Δ*G_Bind_* vdW	−60.87 ± 4.74	−74.21 ± 5.33	−72.51 ± 3.35	−55.96 ± 1.95
Ligand Strain Energy	11.77 ± 3.68	13.96 ± 3.76	6.83 ± 1.39	4.21 ± 0.98

## Supplementary Materials

The following supporting information can be downloaded at: https://www.mdpi.com/article/10.3390/v15071501/s1, Figure S1: RMSD of the protein and ligand during the 100 ns MD simulation of two replicates for the (a,b) 127042987 (c,d) 44577154 (e,f) 127040817; Figure S2: RMSF of the protein residues during the 100 ns MD simulation of two replicates for the (a,b) 127042987 (c,d) 44577154 (e,f) 127040817; Figure S3: Intermolecular interactions mapping during the 100 ns MD simulation of two replicates for the (a,b) 127042987 (c,d) 44577154 (e,f) 127040817; Figure S4: SASA (Solvent Accessible Surface Area) during the 100 ns MD simulation of two replicates for the (a, b) 127042987 (c,d) 44577154 (e,f) 127040817.
