# Supplementary material for: Investigating the Mechanism of Action of Anti-Dengue Compounds as Potential Binders of Zika Virus RNA-Dependent RNA Polymerase
Source: Viruses. 2023 Jul 4;15(7):1501. doi: 10.3390/v15071501 (PMC10384299; doi:10.3390/v15071501)
Supplement: Supplementary file 1 [file viruses-15-01501-s001.zip › viruses-2438717-supplementary.pdf]

Supplementary Materials

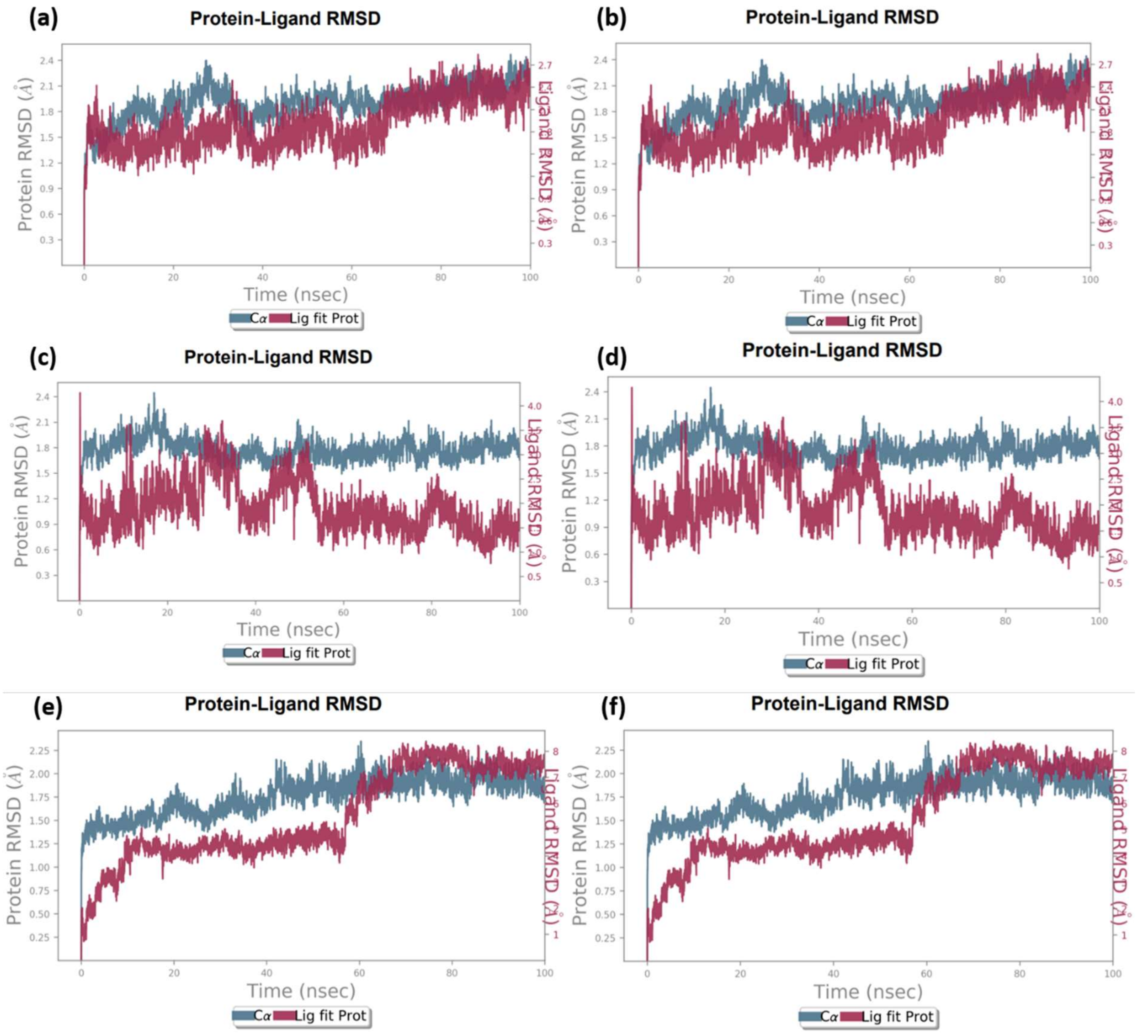

Figure S1. RMSD of the protein and ligand during the 100 ns MD simulation of two replicates for the (a, b) 127042987 (c, d) 44577154 (e, f) 127040817

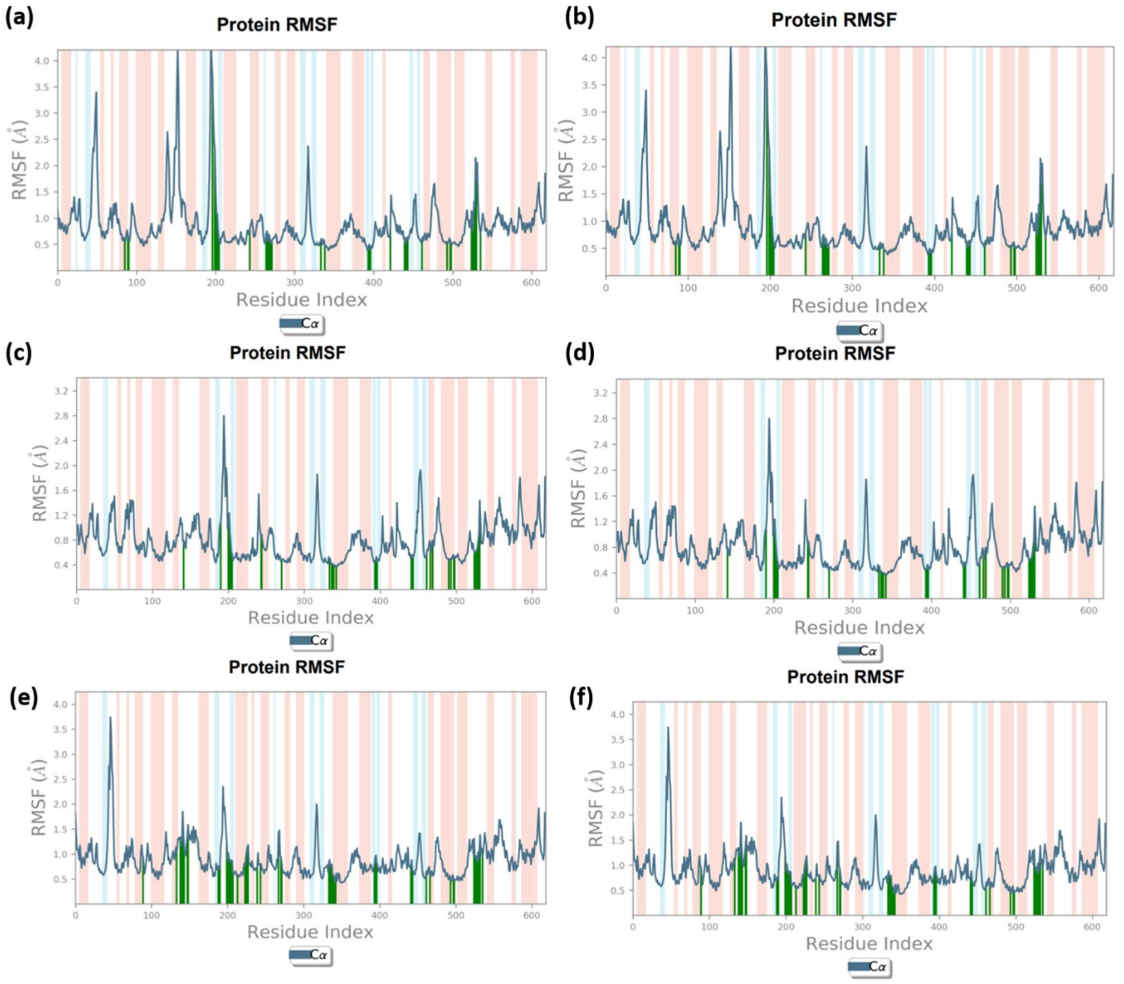

Figure S2. RMSF of the protein residues during the 100 ns MD simulation of two replicates for the (a, b) 127042987 (c, d) 44577154 (e, f) 127040817

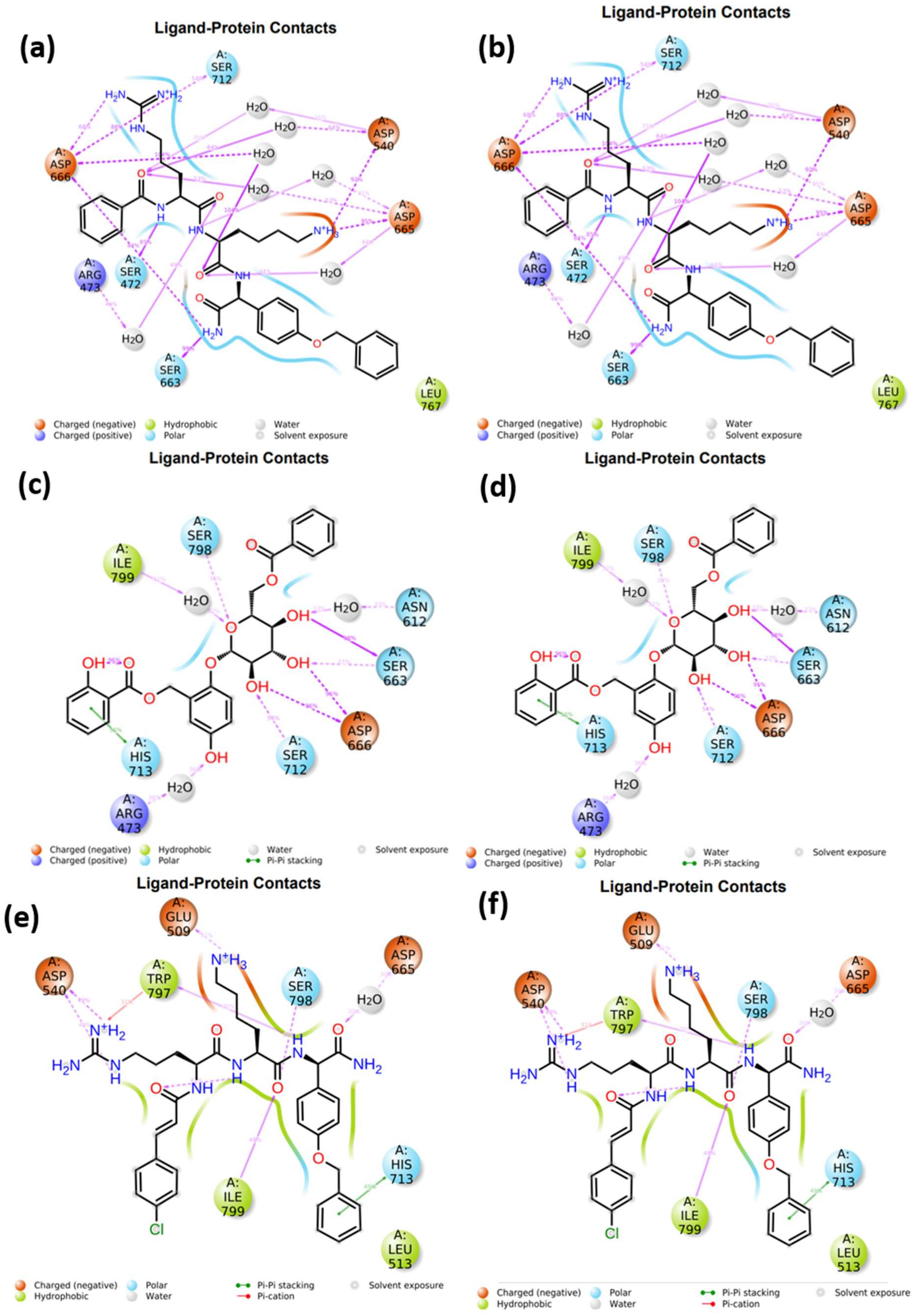

Figure S3. Intermolecular interactions mapping during the 100 ns MD simulation of two replicates for the (a, b) 127042987 (c, d) 44577154 (e, f) 127040817

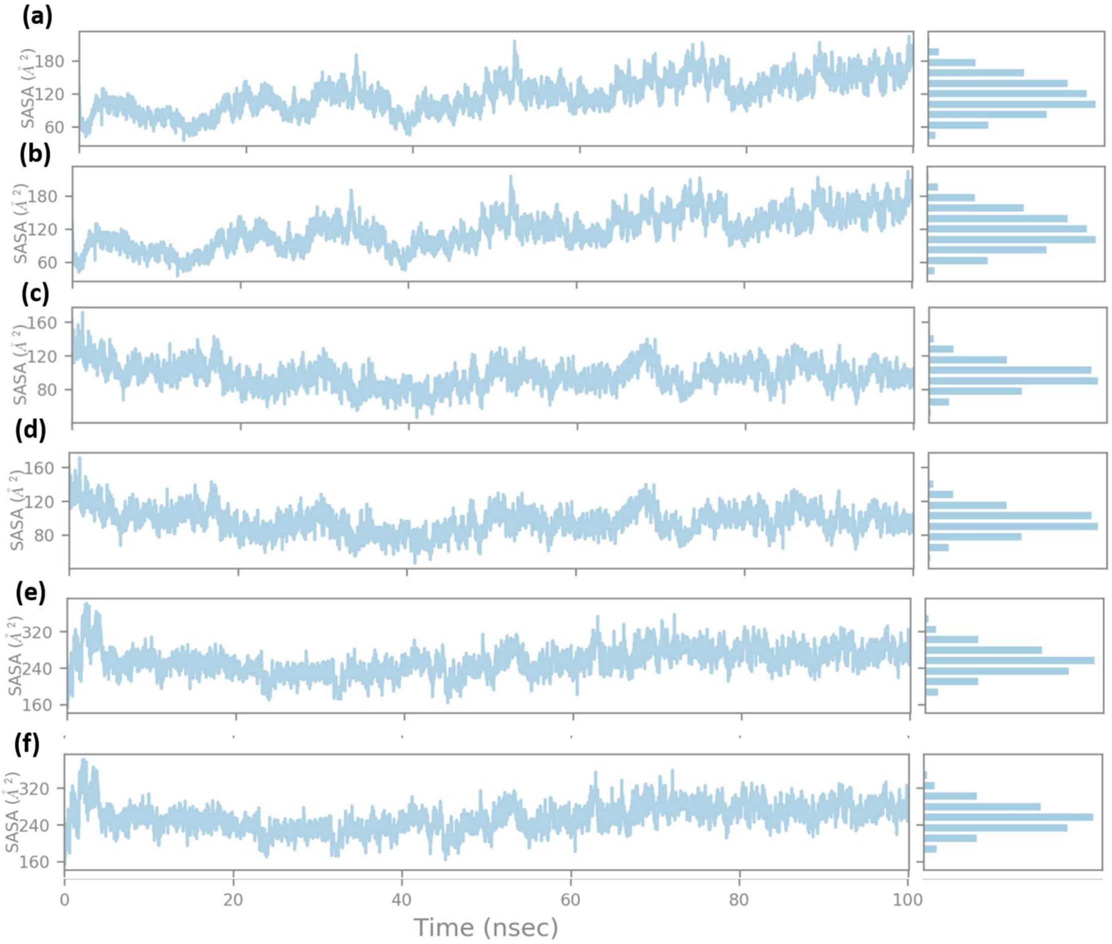

Figure S4. SASA (Solvent Accessible Surface Area) during the 100 ns MD simulation of two replicates for the (a, b) 127042987 (c, d) 44577154 (e, f) 127040817
